# Heat stress increases immune cell function in Hexacorallia

**DOI:** 10.3389/fimmu.2022.1016097

**Published:** 2022-12-22

**Authors:** Shir Eliachar, Grace Ann Snyder, Shany Klara Barkan, Shani Talice, Aner Otolenghi, Adrian Jaimes-Becerra, Ton Sharoni, Eliya Sultan, Uzi Hadad, Oren Levy, Yehu Moran, Orly Gershoni-Yahalom, Nikki Traylor-Knowles, Benyamin Rosental

**Affiliations:** ^1^ The Shraga Segal Department of Microbiology, Immunology, and Genetics, Faculty of Health Sciences, Regenerative Medicine and Stem Cell Research Center, Ben Gurion University of the Negev, Beer Sheva, Israel; ^2^ Department of Marine Biology and Ecology, Rosenstiel School of Marine, Atmospheric, and Earth Science, University of Miami, Miami, FL, United States; ^3^ Department of Ecology, Evolution and Behavior, Alexander Silberman Institute of Life Sciences, Faculty of Science, Hebrew University of Jerusalem, Jerusalem, Israel; ^4^ Ilse Katz Institute for Nanoscale Science and Technology, Ben-Gurion University of the Negev, Beer Sheva, Israel; ^5^ The Mina and Everard Goodman Faculty of Life Sciences, Bar Ilan University, Ramat Gan, Israel

**Keywords:** phagocytosis, comparative immunology, Hexacorallia, innate immunity, heat stress

## Abstract

Climate change induced heat stress has increased coral bleaching events worldwide. Differentially regulated immune genes are one of the primary responses to heat stress suggesting that immune activation is critical. However, the cellular immune mechanisms of coral bleaching is currently unknown, and it is still not known if the immune response documented during heat stress is a consequence of bleaching or is directly caused by the heat stress itself. To address this question, we have used two model system sea anemones (Order: Actiniaria): *Exaiptasia diaphana* and *Nematostella vectensis*. *E. diaphana* is an established sea anemone model for algal symbiont interaction, while *N. vectensis* is an established sea anemone model that lacks the algal symbiont. Here, we examined the effect of increased temperature on phagocytic activity, as an indication of immune function. Our data shows that immune cell activity increases during heat stress, while small molecule pinocytosis remains unaffected. We observed an increase in cellular production of reactive oxygen species with increasing temperatures. We also found that the cellular immune activity was not affected by the presence of the Symbiodiniaceae. Our results suggest that the immune activity observed in heat-stress induced bleaching in corals is a fundamental and basic response independent of the bleaching effect. These results establish a foundation for improving our understanding of hexacorallian immune cell biology, and its potential role in coral bleaching.

## Introduction

Climate change induced heat stress is detrimental to hexacorallian health and is the primary cause of coral reef death throughout the world (1–7). The rise of ocean temperatures has led to the increasing frequency of coral bleaching events, a phenomenon in which endosymbiotic dinoflagellates (family: Symbiodiniaceae) are expelled from the coral host (1, 8). The Symbiodiniaceae reside within coral gastrodermal cells, and are responsible for providing the majority of the coral’s food source (9). During heat stress an increase in reactive oxygen species (ROS) is produced by Symbiodiniaceae, which leads to the damage of both coral host tissues and Symbiodiniaceae membranes (10–15). However, it has also been suggested that Symbiodiniaceae expulsion is unrelated to endosymbiotic ROS (16–19), and may be a result of shifting dynamics within the coral holobiont (20). Regardless, the Symbiodiniaceae and coral host then dissociate *via* several different methods, including host cell apoptosis and host cell-initiated exocytosis of Symbiodiniaceae, among other documented mechanisms (21–27).

Heat stress-induced coral bleaching affects the expression of innate immune genes within the coral (28–30). During early heat stress, the coral host upregulates transcription factors related to innate immune pathways such as ELK-3, NF-kB, and Kruppel-like factors (29, 30). Additionally, many potential immune-like factors, such as TRAF, TNFR, and NOD-like receptors, are upregulated during heat stress (28, 31, 32). Previous research on the effects of increased temperatures has focused on the dysbiosis of the relationship of the Symbiodiniaceae with the hexacorallian hosts and the mechanisms causing bleaching (1, 9, 15, 33–36). However, the functional immune responses independent of the symbiosis with Symbiodiniaceae during heat stress have not yet been teased apart. Given this previous body of research, we hypothesize that the coral immune activation observed in heat stress-induced bleaching is due to a heat stress-specific immune response in Hexacorallia. Further, we hypothesize that hexacorallians without Symbiodiniaceae will still activate their immune system in response to heat stress.

**Figure 1:**
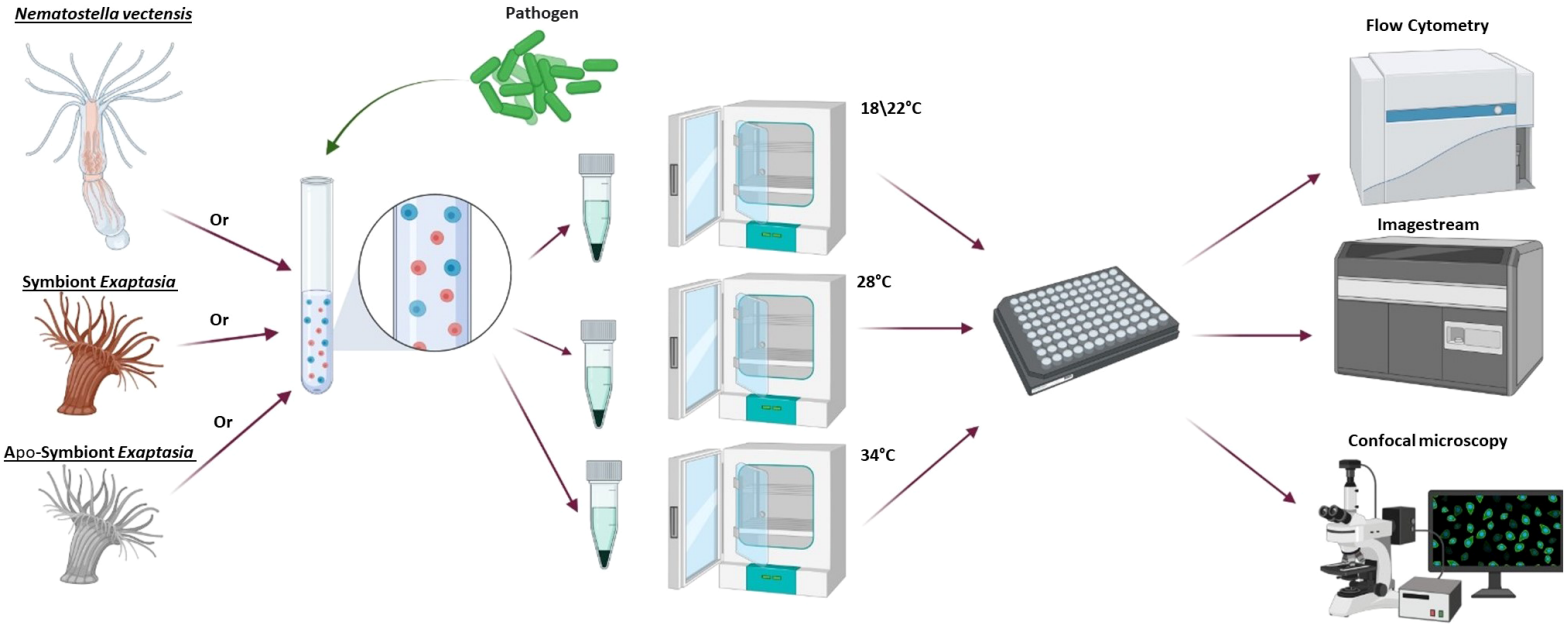
General workflow for heat stress experiments. First, cells were dissociated from one of the following models: *N. vectensis*, symbiotic *E. diaphana* or aposymbiotic *E. diaphana*. The cells were exposed to one of the phagocytic challenges (*S. aureus* or DQ™ ovalbumin) or pinocytosis assay (dextran), then divided into overnight incubators at different temperatures to test the response to heat stress. Following overnight incubations, the cells were concentrated in a 96-well plate and analyzed by imaging flow cytometry and fluorescent confocal microscopy. Diagram was created with BioRender (https://biorender.com/).

To experimentally test our hypothesis, we compared two hexacorallian models, the sea anemones (Order: Actiniaria): *E. diaphana* and *N. vectensis*. While *N. vectensis* lacks Symbiodiniaceae, *E. diaphana* can be raised with and without Symbiodiniaceae. Within each sea anemone population, we performed an overnight phagocytosis assay on isolated cells to test the cellular immune function under heat-stress, compared to pinocytosis under heat stress (**Figure 1**). Previously, we functionally characterized phagocytosis and the phagocytic cells in both the sea anemone, *N. vectensis* and the stony coral, *P. damicornis* (37). Specialized immune cells use phagocytosis as the primary mechanism to engulf and degrade target particles such as pathogens and damaged cells (38, 39). Phagocytosis is mediated by a rapid rearrangement of the actin cytoskeleton, in which pseudopodia extensions engulf the target. Once engulfed, the target will fuse with a phagolysosome for degradation (40). On the other hand, pinocytosis is a type of endocytosis in which cells ingest extracellular fluid and small molecules. We've previously observed similarities in phagocytic mechanisms between the two species (37), strengthening the establishment of *N. vectensis* as a model for immune studies in Hexacorallia.

Here we show that the immune system of sea anemones is activated under short-term heat stress. Moreover, this mechanism is not dependent on the presence of Symbiodiniaceae. Additionally, we show that in short-term heat stress ROS production is carried out by the sea anemone cells regardless of having Symbiodiniaceae. Finally, we suggest that the immune activation in short term heat stress could be a conserved mechanism in Hexacorallia.

## Methods

### Animal husbandry

*N. vectensis* individuals were generously provided by Prof. Tamar Lotan from Haifa University. Animals were maintained at the mariculture room at the Regenerative Medicine and Stem Cell Research Center, Ben Gurion University (approved by the Israel ministry of agriculture and university biosafety committee). All artificial seawater (ASW) used to maintain the animals for this study was made with Red Sea Salt (Red Sea). Both *N. vectensis* and *E. diaphana* were fed freshly hatched Artemia 3-4 times a week. *N. vectensis* was maintained at 18°C in 14 ppt ASW with a pH ranging from 8-8.6. Symbiotic *E. diaphana* (harboring clade B Symbiodiniaceae) and aposymbiotic *E. diaphana* (*E. diaphan*a lacking Symbiodiniaceae) were maintained at 22°C in 35 ppt ASW with a pH ranging from pH 8-8.6 (41, 42). The symbiotic *E. diaphana* were raised in a light/dark cycle of 12 hours, while aposymbiotic *E. diaphana* were raised in 24 hours of dark. Three days before the start of the experiments, *N. vectensis* and *E. diaphana* were starved and isolated within plastic petri dishes.

### Cell dissociation

The same cell dissociation protocol was used for both *N. vectensis* and *E. diaphana*. Cell suspensions were produced from a minimum of 5 animals per experiment, using mechanical dissociations with a sterile razor blade and filtration through 100 µm and 40 µm cell strainers (37, 43, 44). A syringe plunger was used to help facilitate filtering. The culture media used was made of L-15 supplemented with 2% heat-inactivated fetal bovine serum (FBS), and 20 mM HEPES. It was then brought to 1.42 × PBS molarity for *N. vectensis* or 3.3 × PBS for *E. diaphana* using calcium- and magnesium-free 10 × PBS and supplemented with 0.05% NaN_3_ to reduce contamination. The entire cell dissociation process was done on ice to lower cell metabolism and minimize cell damage. Cells were then washed by centrifugation at 500 × *g* at 4°C for 5 minutes and resuspended in 1 ml of culture media.

After the cell dissociation, the cells were counted on an Automated Cell Counter (TC20™- Bio-Rad). Then the cells were used in either phagocytosis or pinocytosis assays. Cells of *N. vectensis* and *E. diaphana* were plated in 96 well U-shaped plates, with 100,000 cells/well in 200 µl of staining culture media.

### Phagocytosis and pinocytosis assays

Two types of phagocytic assays were performed overnight as previously published (37). The first was a bacterial challenge of 15 µg/ml of inactive *Staphylococcus aureus* particles (pHrodo™ Green *S. aureus* Bioparticles™ Conjugate for Phagocytosis; Thermo Fisher Scientific). The pHrodo conjugation to *S. aureus* causes green fluorescence emission when engulfed within low-pH vesicles. In the second assay, DQ™ ovalbumin was used at 15 µg/ml (ThermoFisher Scientific, D12053). This assay measures the protease activity of phagocytosis. We previously modified this established protocol for hexacorallian phagocytic cells (37). In short, the DQ™ ovalbumin is self-quenching and labeled with BODIPY dye (boron-dipyrromethene). Only upon engulfment and fusion with a lysosome will it be hydrolyzed into peptides and emit a green fluorescence (45). To measure pinocytosis, fluorescently tagged dextran molecules, Fluorescein Isothiocyanate – Dextran, were used at 0.65 µg/ml. Dextran is a complex sugar molecule derived from bacteria used as an assay for pinocytosis (Fluorescein Isothiocyanate - Dextran; molecular weight 500,000 MW; Sigma-Aldrich). For phagocytic activity and pinocytosis assays both *N. vectensis* and *E. diaphana* cells were incubated with the reagents overnight. For experiments examining two temperatures DQ™ ovalbumin, pHrodo™ Green *S. aureus* Bioparticles™, and Dextran were used. For the heat ramp assays pHrodo™ Green *S. aureus* Bioparticles™, and Dextran were used.

### Flow cytometry analysis

To differentiate between cells and the phagocytosis reagents (i.e. pHrodo™ Green *S. aureus* Bioparticles), cells were pre-labeled with CellTrace™ Far Red (1:1000) for 30 min. Phagocytic activity was detected using a flow cytometer (NovoCyte flow cytometer, Acea). pHrodo™ Green, DQTM ovalbumin, and Fluorescein Isothiocyanate – Dextran were all analyzed by flow cytometry using a 488 nm laser and detected using a 530/30 nm filter. CellTrace™ Far Red (excitation with 630 nm laser and detection using 675/30 nm filter) was used to detect cells (37). Data analysis was conducted using NovoExpress software.

For two temperature comparisons at least three experiments were performed. For heat ramp assays, each temperature was at least in two independent experiments. In addition, ambient temperature was used in each of the experiments for normalization. For comparing between the effect of two temperatures in phagocytosis and pinocytosis, statistical analysis (Student t-test) was performed in Graphpad Prism for Windows, version 9.4.1.

In order to compare the effect of increasing temperature on phagocytosis and pinocytosis in *N. vectensis* and *E. diaphana*, we normalized the percentage of phagocytosis and pinocytosis to ambient temperature using Excel software. Next, we used Mixed effects analysis, using the restricted maximum likelihood method (REML), by Graphpad Prism for Windows, version 9.4.1 (**Supplementary Table 1**).

### Heat stress at variable temperatures

To test the effects of heat stress, cell suspensions undergoing the above phagocytosis assay or pinocytosis assay were incubated in ambient or elevated temperatures. For *N. vectensis* cells, an ambient temperature of 18°C (46, 47) and elevated temperatures of 21°C, 24°C, 28°C, and 34°C were used. *N. vectensis* has been observed to tolerate temperatures up to 30°C, although it is typically reared at 18°C in the laboratory (48, 49). *E. diaphana* cells were kept at an ambient temperature of 22°C (50), and elevated temperatures of 25°C, 27°C, 30°C, 32°C, or 34°C. *E. diaphana* are naturally found in tropical waters and could be reared in laboratory temperatures between 20-26°C *(General Aiptasia husbandry - Weis Lab*; 51). We observed tissue degradation at 34°C after performing a heat tolerance experiment on the whole animal level of aposymbiotic *E. diaphana*, making it our highest temperature for testing (**Figure S1**).

### Detection of reactive oxygen species

To test whether cells increase ROS production when exposed to high temperature, cells were exposed to 11.2 ng/ml of DCFDA-ROS Detection Cell-Based Assay Kit (Cayman). Cells from *N. vectensis*, symbiotic *E. diaphana*, and aposymbiotic *E. diaphana* were incubated overnight in the variable temperatures. The next morning, following cell counting and distribution of 50,000 cells/well in a 96 U-shaped plate, cells were incubated for 45 minutes in the DCFDA-ROS Detection Cell-Based Assay at ambient temperatures. Detection of DCFDA-ROS was then measured by flow cytometry using a 488 nm laser and detected on a 530/30 nm filter. The signal measurement was compared to the unstained controls. For comparing the effect of temperature on ROS enrichment in *N. vectensis* and *E. diaphana*, we used an unpaired t-test, using Microsoft Excel software.

### Symbiodiniaceae measurement in medium

To measure the expulsion of symbiotic Symbiodiniaceae into the surrounding water, we incubated the symbiotic *E. diaphana* overnight in 24 well plates, at 2.5 ml of 35 ppt ASW. We incubated animals in different temperature including ambient temperature of 22°C (50) and elevated temperatures of 25°C, 28°C, 30°C, 32°C, or 34°C. Following overnight incubation, we measured the far-red autofluorescence of the Symbiodiniaceae by sampling 0.5 ml out of the 2.5 ml of the surrounding medium using FACS analysis. For comparing the effect of temperature on algae expulsion in *E. diaphana*, we used an unpaired t-test, using Microsoft Excel software.

### Imaging flow cytometry

After the overnight incubations for both ambient and heat stress treatments, cells were analyzed using ImageStream X Mark II Imaging flow cytometer (Amnis, Co., Seattle, WA, USA) with a 40×/0.75 objective. As previously done in (37) channels representing bright-field, green channel (excitation laser 488nm, filter band 533/55nm), and far-red channel (excitation laser 642nm, filter band 702/85nm), were used to record 10,000 cells for each sample. IDEAS^®^ software was then used to quantify the measurements of cells that were in focus, single cells, and had a fluorescent signal.

### Confocal microscopy

Cells from representative treatments were transferred into 500 µL of staining culture media, centrifuged for 5 minutes at 500 × *g* at 4°C, and resuspended in 50 µL of staining culture media. The resuspended cells were transferred to 384 glass bottom imaging plate (cell culture microplate 384 well, CLEAR^Ⓡ^, Poly-L-lysine, cellcoat^Ⓡ^, Greiner bio-one). Images were acquired using a 20 × objective on a ZEISS LSM900 confocal microscope and analyzed using ZEISS ZEN-black software.

### Immune gene identification and analysis

To identify upregulated immune genes in *N. vectensis* during heat stress, we used a previously published gene expression data set (52). This study sought to evaluate the impact of abiotic stressors such as heat, salinity and, UV light on venom production of distinct populations along the East coast of North America of *N. vectensis*. The raw reads for stress and control treatments from two populations, Massachusetts (MA) and North Carolina (NC) were used in our study. Raw reads were mapped with STAR v.2.7.5 (53) to *N. vectensis* gene models and counted with Featurecounts (54). Differential expression analysis of the two populations was performed using the DESeq2 v1.6.3 R package (55). The raw counts tables were imported into R Studio and normalized using the median of ratios (56) with the “estimateSizeFactors” function in DESeq2. Genes with over two-fold changes (log2 fold change> 1) and a False Discovery Rate (FDR) cut off of FDR ≤ 0.05 were considered as significantly differentially expressed genes.

To characterize potential immune genes among significant differentially expressed up-regulated genes, Blastp with *e* value ≤1e-5 (57) and HMM version 3.3.2 with *e* value ≤1e-5 (hmmer.org) searches were performed against a custom database of immune up-regulated genes in abiotic stress found in previous studies on Anthozoa. This custom database was built with protein sequences downloaded from the RefSeq nr database (58) from the species *Acropora digitifera*, *Acropora millepora*, *Acropora palmata*, *Actinia tenebrosa*, *E. diaphana*, *N. vectensis*, *Orbicella faveolata*, *Paramuricea clavata*, *P. damicornis* and *Stylophora pistillata* for pathways labeled with key words including: “ tumor necrosis factor receptor”, “apoptotic”, “enzymatic”, “NF-kappa-B”, “HLF”, “nod-like receptor” and “inflammatory”.

## Results

### Immune gene expression in the *N. vectensis* model in stress

To test whether hexacorallians without Symbiodiniaceae activate their immune system in response to heat stress, we examined previously published data on gene expression in *N. vectensis* under a combination of abiotic stressors, including heat stress (52). We found similar immune gene homologs between *N. vectensis* and corals that were upregulated in response to heat stress (52; **Table 1**). These genes include TNF receptor-associated factors, Caspase-8, Hepatic Leukemia Factor, and the inflammatory marker of Kruppel-like Factors.

**Table 1:** Eight upregulated genes were identified in searches.

Gene Model	Annotation	Log2Fold Change*
NVE10794	TNF receptor associated factor	2.64
NVE12824	TNF receptor associated factor	3.24
NVE1476	TNF receptor associated factor	1.81
NVE26090	Caspases	2.12
NVE9681	Caspases	2.55
NVE7846	Inflammatory Kruppel-like factor	1.1
NVE8107	Hepatic leukemia factor	1.88
NVE5408	Hepatic leukemia factor	5.22

Columns include: Gene model=identifier for upregulated model gene in *Nematostella vectensis.* Annotation=protein domains found in genes identified. Log2Fold Change= log2 fold change value of gene identified when compared to control treatment. * The log2 fold change values in the MA population were similar.

### Phagocytic activity increases in the *N. vectensis* model

**Figure 2:**
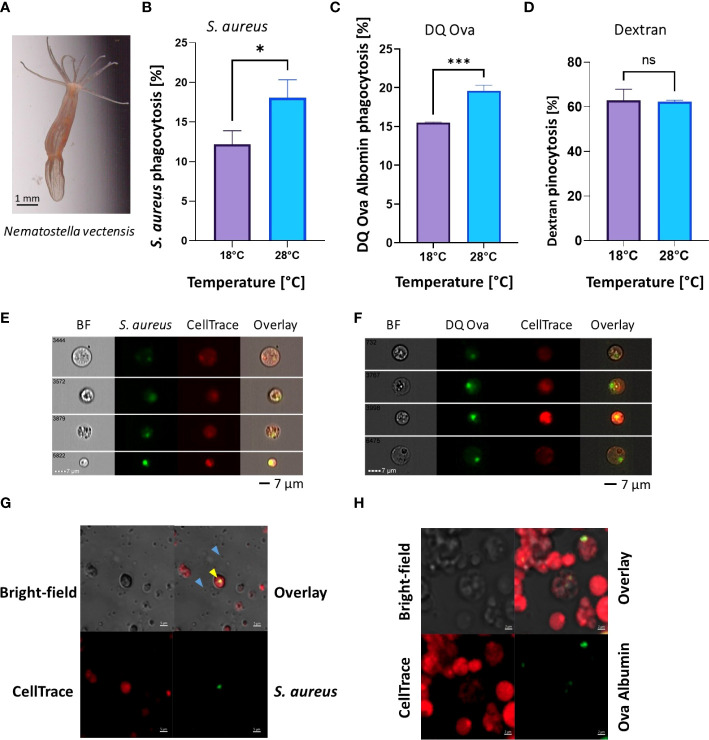
Phagocytic activity increases in heat-stress conditions, while pinocytosis remains unchanged in *N. vectensis*. **(A)** *N. vectensis* imaged by ZEISS Axiocam 208 Color Microscope Camera. **(B, C)** Phagocytosis of *S. aureus* bioparticles and DQ ovalbumin, respectively, under heat stress conditions of 28°C, increased significantly from the ambient treatment (18°C). **(D)** Pinocytosis of dextran under heat stress did not significantly change between ambient and heat stress conditions. **(E)** ImageStream analysis of cells that have engulfed *S. aureus* particles. The green fluorescence is emitted from the *S. aureus* particles, but only when in low-pH vesicles; red fluorescence is from the CellTrace cell stain. **(F)** ImageStream analysis of cells that have engulfed DQ ovalbumin. The green fluorescence is emitted from the DQ ovalbumin conjugation with BODIPY, but only once hydrolysis into single peptides has occurred. A Student’s T-Test analysis was performed on the phagocytic and pinocytic assays between the ambient and elevated temperatures. **(G)** Confocal images of *N. vectensis* cells challenged with inactive pHrodo™ *S. aureus* bioparticles (blue arrows), with cells having internalized pH-activated bioparticles in green (yellow arrow). **(H)** Confocal images of *N. vectensis* cells challenged with DQ ovalbumin, upon protease activity DQ ovalbumin fluoresces in green. A Student’s t-test analysis was performed on the phagocytosis and the pinocytosis assays comparing the ambient and elevated temperatures. P-value legend: ns, Not significant, *: p <0.05, ***: p < 0.001. Scale bar represents standard deviation (SD).

To test the effects of elevated heat on the immune system at the functional level we used two phagocytic cell assays: pHrodo Green *S. aureus* bioparticles and DQ™ ovalbumin protein, or dextran as a pinocytosis control (previously established in 37). In *N. vectensis*, a significant increase in the percentage of active phagocytic cells was detected in the elevated temperature (28°C) in comparison to the ambient temperature (18°C) (Student’s t-test, * = p <0.05; **Figures 2A, B**). In contrast, overnight exposure with the pinocytosis stimulant dextran showed no significant change between the ambient (18°C) and elevated (28°C) temperatures (**Figure 2D**). For validation of *N. vectensis* cellular phagocytosis, ImageStream analysis (**Figures 2E, F**) and confocal analysis (**Figures 2G, H**) showed that the phagocytic signal is localized within the cells.

### Phagocytic activity during heat stress in aposymbiotic and symbiotic *E. diaphana* model

**Figure 3:**
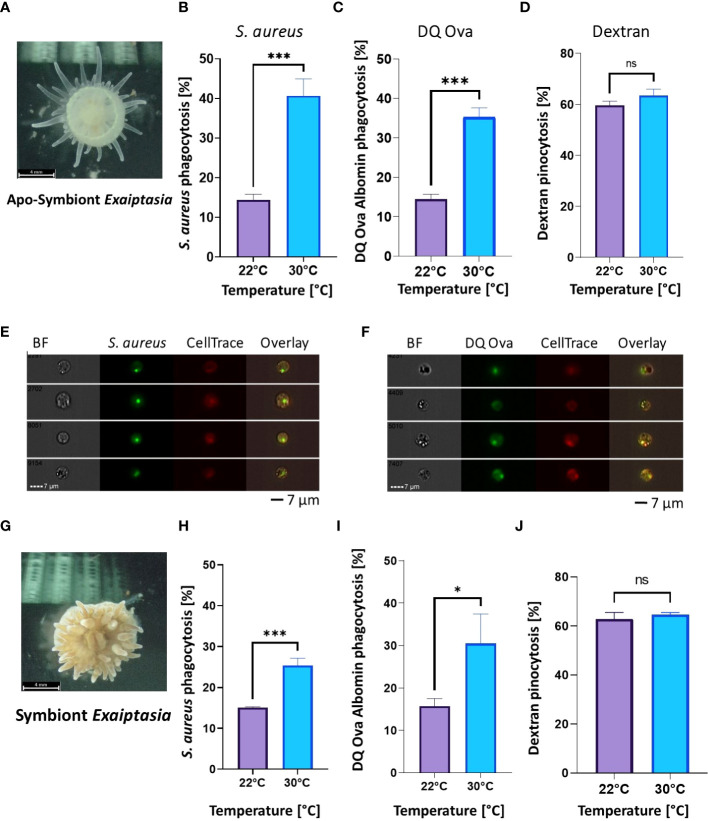
Phagocytic activity increases in heat stress conditions, while pinocytosis remains unchanged in both aposymbiotic and symbiotic *E. diaphana*. **(A)** Aposymbiotic *E. diaphana* imaged by ZEISS Axiocam 208 Color Microscope Camera. **(B, C)** Phagocytic cell activity in aposymbiotic *E. diaphana* of *S. aureus* particles **(B)** and DQ ovalbumin **(C)** is significantly higher under heat stress conditions 30°C, compared to ambient conditions of 22°C. **(D)** Pinocytosis of dextran in aposymbiotic *E. diaphana* under normal and heat stress conditions are not significantly different between ambient (22°C) and thermal stress conditions (30°C). **(E, F)** Phagocytosis images by Image stream analysis for aposymbiotic *E. diaphana* of *S. aureus* particles **(E)** and DQ ovalbumin **(F)**. **(G)** Symbiotic *E. diaphana* imaged by ZEISS Axiocam 208 Color Microscope Camera. **(H, I)** Phagocytic activity in symbiotic *E. diaphana* of *S. aureus* particles **(H)** and DQ™ ovalbumin **(I)** is significantly higher under heat stress conditions (30°C), compared to ambient (22°C). **(J)** Pinocytosis of dextran in symbiotic *E. diaphana* under normal and heat stress conditions are not significantly different. A Student’s t-test analysis was performed on the phagocytic and the pinocytosis assays comparing the ambient and elevated temperatures. P-value legend: ns, Not significant, *: p <0.05, ***: p < 0.001. Scale bar represents standard deviation (SD).

To further test whether the effects of heat stress on phagocytosis were conserved in Hexacorallia harboring Symbiodiniaceae we employed the model *E. diaphana*, which is capable of living with and without Symbiodiniaceae. Similar to *N. vectensis*, regardless of the presence of Symbiodiniaceae, phagocytic cell activity was significantly higher after overnight exposure to a moderate heat stress (30°C) compared with ambient temperatures (22°C) (**Figures 3A, B, F, G**), while pinocytosis remained unaffected (**Figures 3C, H**).

### Immune function changes in variable temperatures of heat stress

**Figure 4:**
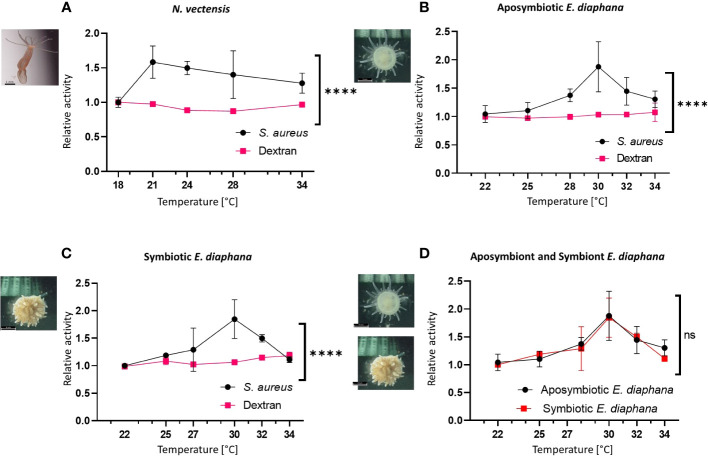
Immune activation vs pinocytosis at increased temperatures in hexacorallian models. **(A)** The relative change in the flow cytometry percentage of activity in the *N. vectensis* phagocytic cells (normalized to ambient temperature) significantly elevated with heat, compared to the control pinocytosis of dextran. **(B, C)** The relative change in the flow cytometry percentage of activity in the aposymbiotic *E. diaphana* **(B)** and symbiotic *E. diaphana* **(C)** phagocytic cells (normalized to ambient temperature) significantly elevated with heat, compared to the control pinocytosis of dextran. **(D)** The relative percentage change in different temperatures of the *S. aureus* phagocytosis in the aposymbiotic and the symbiotic *E. diaphana* phagocytic cell is similar. Control temperature for the *N. vectensis* model is 18°C and 22°C is the control temperature for the *E. diaphana* models. Mixed effects analysis was performed on the phagocytosis and the pinocytosis series for each animal model **(A–C)**, and between the two *E. diaphana* models **(D)**. Mixed effects analysis, using the model of restricted maximum likelihood method (REML; detailed in **Supplementary Table 1**). P-value between treatments: ns, Not significant, ****: p < 0.0005. Scale bar represents SD.

To test the effects of variable temperatures on immune function we applied phagocytic and pinocytic assays to *N. vectensis*, and aposymbiotic or symbiotic *E. diaphana* at different elevated temperatures. *N. vectensis* cells had a significant increase in their phagocytic activity of pHrodo™ Green *S. aureus* Bioparticles (**Figure 4A**). Response peaked at 21°C and incrementally declined while still being above ambient temperature. In contrast, there was no significant change in the pinocytic activity in variable temperatures compared to 18°C (**Figure 4C**). In *E. diaphana*, similar phagocytic responses were observed in both aposymbiotic and symbiotic individuals. The greatest relative change compared to the ambient treatment occurred at 30°C and declined afterwards, with 34°C reaching almost to the level of ambient temperature at 22°C. As in *N. vectensis*, the relative pinocytic activity did not significantly change across varying degrees of heat stress (**Figures 4B, C**). Interestingly, when overlaying the relative phagocytic activity of symbiotic and aposymbiotic *E. diaphana* there is no difference between the two reactions, suggesting that Symbiodiniaceae doesn’t affect the immune regulation in heat.

### Elevated temperatures increase cellular ROS production as well as algae expulsion

**Figure 5:**
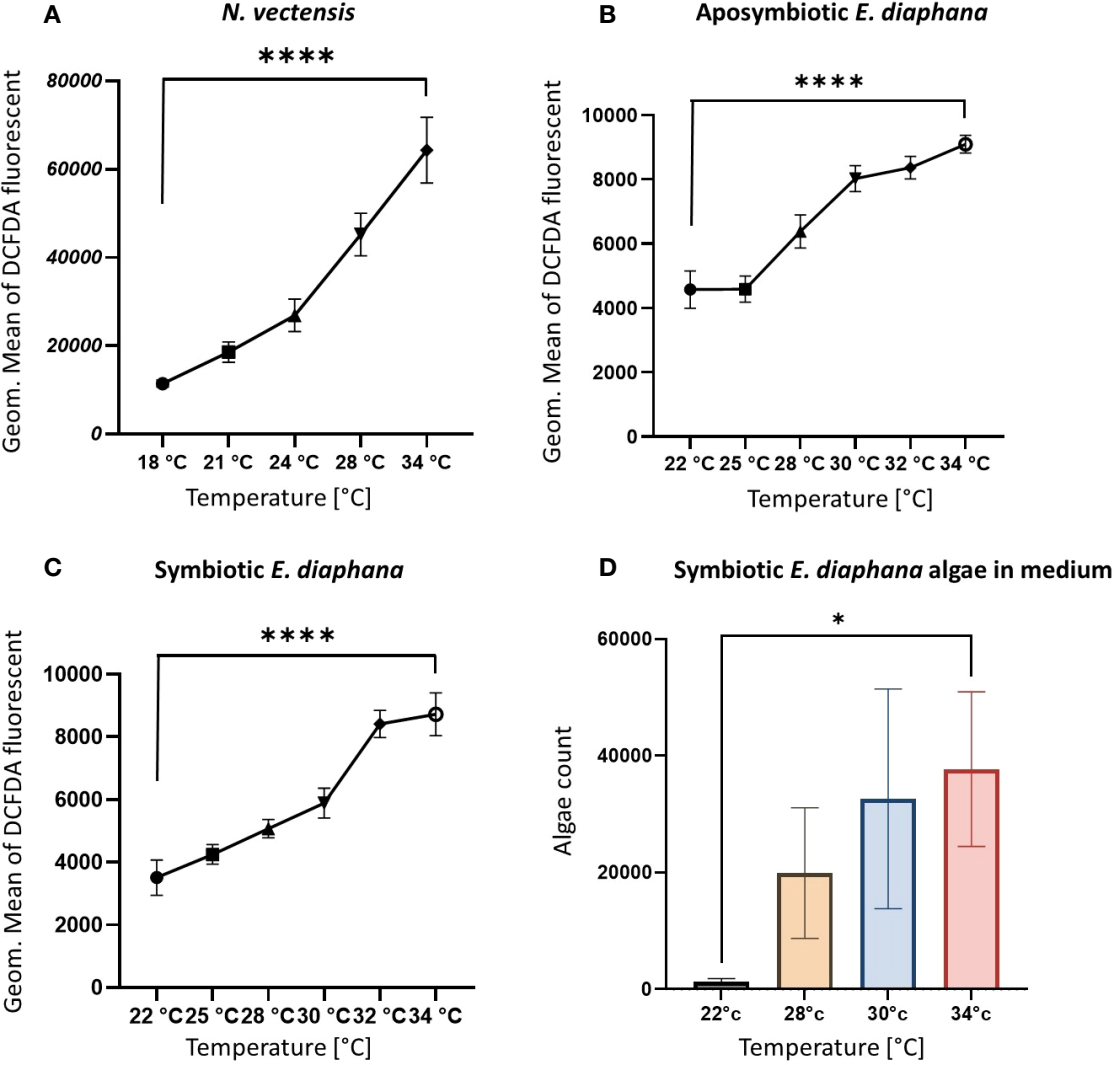
Parameters of increased heat response validation. **(A, B)** The levels of ROS increases on the cellular level in elevated temperatures in the *N. vectensis* model **(A)** aposymbiotic **(B)** and symbiotic **(C)** *E. diaphana*, measured by flow cytometry fluorescence of the DCFDA marker for ROS, in each cell. Measured by Geometric mean of the green fluorescence of the cells. **(D)** Counts of expelled algae from *E. diaphana* due to heat stress in 2.5 ml of media. Control temperature for the *N. vectensis* model is 18°C and 22°C is the control temperature for the *E. diaphana* models. Unpaired t-test analysis was performed on the ROS enrichment and algae count between the ambient temperature and 34°C. P-value legend: *: p <0.05, ****: p < 0.0005 (**Supplementary Table 1**). Scale bar represents SD.

To test whether heat stress leads to an increase in ROS production independent of the symbiotic Symbiodiniaceae, we used DCFDA markers for intracellular ROS in cells from *N. vectensis*, symbiotic, and aposymbiotic *E. diaphana*, that were exposed to a range of temperatures. Intracellular ROS was significantly increased in elevated temperatures, compared to ambient conditions (**Figure 5A**). Similarly, both aposymbiotic and symbiotic *E. diaphana* showed increases in intracellular ROS under thermal stress conditions compared to the ambient temperature (**Figures 5B, C**).

To validate that the temperature conditions were inducing bleaching, we measured the expulsion of symbiotic Symbiodiniaceae from *E. diaphana* into the surrounding water after overnight incubation at the various temperatures. The number of Symbiodiniaceae cells in the medium was observed to have increased significantly compared to ambient conditions (**Figure 5D**), indicating the expulsion of Symbiodiniaceae from the host cells.

## Discussion

### Immune cellular function activity is heightened in heat induction

Previous heat stress gene expression studies on many different hexacorallians have shown differential gene expression of immune genes, suggesting immune activation is occurring (28–32, 59). Here, we found that during heat stress phagocytic activity increased, indicating that heat stress can increase the immune cell activity. Further, pinocytosis was not affected by the heat stress, indicating that within these assays, normal metabolic function was maintained (**Figures 2**-**4**). Phagocytosis and pinocytosis processes are functionally different in hexacorallians, where phagocytosis is executed by a specific subpopulation of cells and not the majority of the cells like what is observed in pinocytosis (**Figures 2**, **3**) (37, 60). Previous studies on heat stress effects in aposymbiotic and symbiotic *E. diaphana* found an upregulation of immune genes during the first 12 hours, followed by a decrease to near baseline levels (59). Similarly, our overnight assays of 12 hours showed significant increase in immunological function in both symbiotic and aposymbiotic animals. Our results may indicate that heat stress in Hexacorallia does not reduce immunocompetence, but rather increases it, perhaps even to a potentially harmful level. Hyperimmune responses are not well characterized in invertebrates, but in vertebrates, including humans have been documented (61–63). Future research on measuring and characterizing this potential hyperimmune response in corals may be important to our understanding of the different disease and heat stress pathologies that have been previously observed (64).

### Immune activation during heat stress is not a by-product of bleaching

Previous observations of differential gene expression of immune genes suggested immune activity during heat stress in Hexacorallia was due to the process of bleaching (28–32). Here we show that the immune activation in heat stress occurs regardless of the presence of the symbiotic Symbiodiniaceae. We were able to tease apart if the increase in phagocytosis was linked to the presence of Symbiodiniaceae, and found that the increase of phagocytosis was independent of the algal symbiosis, suggesting that the immune response and bleaching response can be uncoupled. This shows that immune activation during heat stress is not a byproduct of heat-induced bleaching. Moreover, the aposymbiont and symbiont models of *E. diaphana* respond in a similar way to heat stress (**Figure 4D**) (59). When we tested for ROS production (15), we observed that it is also produced by the animal cells independently of the presence of symbiotic algae (**Figures 5A-C**). Our observations support the hypothesis that the ROS production in heat-induced bleaching is done not only by the algae but also by hexacorallian cells. This supports previous suggestions that ROS production is not solely a marker for photosynthetic stress (65). Together, these results suggest that there is an underlying immune response to heat stress, and it is independent of the symbiotic dinoflagellate algae, Symbiodiniaceae.

### Immune activation in heat could be a conserved evolutionary mechanism

Immune system response upon heat induction is well documented across vertebrates. Upon infection, warm blooded organisms will initiate a febrile response, while cold blooded organisms will raise their body temperature by behavioral means (66–74). In the case of marine invertebrate organisms, they are dependent on environmental temperature fluctuations (75). Warming water temperatures or heat wave events have been linked to an increase in pathogenic *Vibrio* sp. abundance in several locations (76–79). During heat waves, the microbial population may shift in favor of more opportunistic pathogenic species and can lead to higher mortality due to infection (80). It is plausible that marine invertebrates evolved means to respond and combat these pathogenic stressors in heat, by activating their immune system (81–84). However, there are also examples of immune activity reduction in marine invertebrates in long-term exposure to heat stress (85–88). These examples show the importance of the temporal aspects of the immune response and highlight the need for further comparisons between short-term heat stress immune activation and long-term/chronic heat stress immune reduction. While our study only examined a heat stress exposure over 12 hours, it is important to note that this was comparable to the gene activity observed in *E. diaphana* previously (59). Lastly, our experiments show that temperature levels used in heat stress exposures are also important when measuring levels of immune activation (**Figure 4**). Further investigation into the temperature duration and ranges in which immune activity occurs will help our understanding of the limits under which it can function in hexacorallians.

### Conclusions

Within the hexacorallian models *N. vectensis* and *E. diaphana* we show that during heat stress there is increased phagocytic activity indicative of increased immune activity. Using aposymbiont and symbiont models, we show that this immune activity is independent of Symbiodiniaceae presence. We also show that there is significant ROS production in hexacorallian cells during heat induction. Finally, this work proposes that the immune activation by heat stress induced bleaching, previously suggested in corals, is a basic mechanism in Hexacorallia that might be independent of the bleaching itself, or possibly even contributes to bleaching.

## Data availability statement

The original contributions presented in the study are included in the article/**Supplementary Material**. Further inquiries can be directed to the corresponding authors.

## Author contributions

NT-K, GS and BR conceived and designed the research study. SE performed the phagocytosis experiments. ST, OG-Y, UH, and AO assisted with microscopy and image cytometry. SE, ST, ES, SB, BR, and OG-Y ran the experiments. AJ-B, TS, YM performed gene analysis. OL produced the aposymbiont animals. All authors assisted with the writing and editing. All authors contributed to the article and approved the submitted version.
