# Supplementary material for: Heat stress increases immune cell function in Hexacorallia
Source: Front Immunol. 2022 Dec 22;13:1016097. doi: 10.3389/fimmu.2022.1016097 (PMC9815446; doi:10.3389/fimmu.2022.1016097)
Supplement: Supplementary file 2 [file Image_1.pdf]

Supplementary Figure1

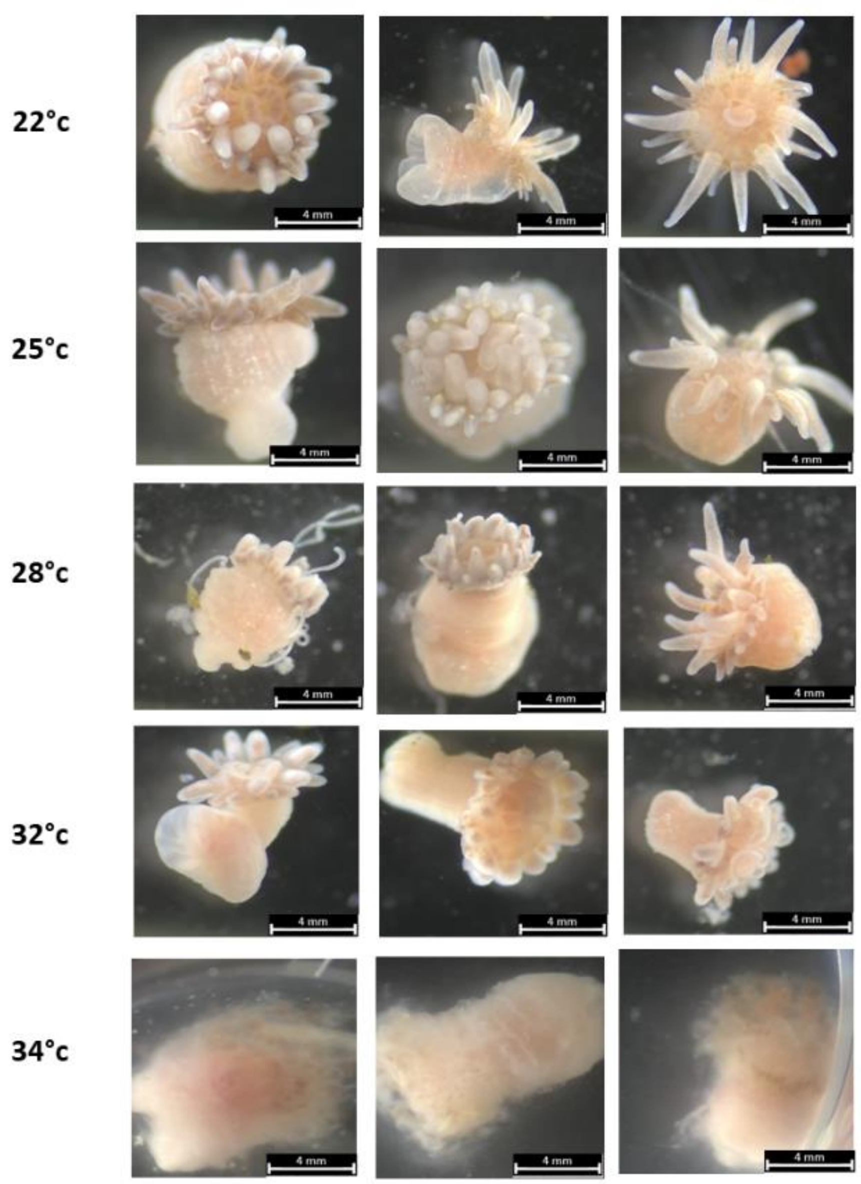

Figure S1: Physiology of *E. diaphana* tissue under heat stress. Examples of aposymbiont *E. diaphana* response to heat stress in variable temperatures. Tissue disintegration was observed after exposure to 34°C.
